# I Just Ran a Thousand Analyses: Benefits of Multiple Testing in Understanding Equivocal Evidence on Gene-Environment Interactions

**DOI:** 10.1371/journal.pone.0125383

**Published:** 2015-05-27

**Authors:** Vera E. Heininga, Albertine J. Oldehinkel, René Veenstra, Esther Nederhof

**Affiliations:** 1 University of Groningen, University Medical Center Groningen, Department of Psychiatry, Interdisciplinary Center Psychopathology and Emotion regulation, Groningen, the Netherlands; 2 University of Groningen, Department of Sociology, Groningen, the Netherlands; Radboud University, NETHERLANDS

## Abstract

**Background:**

In psychiatric genetics research, the volume of ambivalent findings on gene-environment interactions (G x E) is growing at an accelerating pace. In response to the surging suspicions of systematic distortion, we challenge the notion of chance capitalization as a possible contributor. Beyond qualifying multiple testing as a mere methodological issue that, if uncorrected, leads to chance capitalization, we advance towards illustrating the potential benefits of multiple tests in understanding equivocal evidence in genetics literature.

**Method:**

We focused on the interaction between the serotonin-transporter-linked promotor region (5-*HTTLPR*) and childhood adversities with regard to depression. After testing 2160 interactions with all relevant measures available within the Dutch population study of adolescents TRAILS, we calculated percentages of significant (*p* < .05) effects for several subsets of regressions. Using chance capitalization (i.e. overall significance rate of 5% alpha and randomly distributed findings) as a competing hypothesis, we expected more significant effects in the subsets of regressions involving: 1) interview-based instead of questionnaire-based measures; 2) abuse instead of milder childhood adversities; and 3) early instead of later adversities. Furthermore, we expected equal significance percentages across 4) male and female subsamples, and 5) various genotypic models of 5-*HTTLPR*.

**Results:**

We found differences in the percentages of significant interactions among the subsets of analyses, including those regarding sex-specific subsamples and genetic modeling, but often in unexpected directions. Overall, the percentage of significant interactions was 7.9% which is only slightly above the 5% that might be expected based on chance.

**Conclusion:**

Taken together, multiple testing provides a novel approach to better understand equivocal evidence on G x E, showing that methodological differences across studies are a likely reason for heterogeneity in findings - but chance capitalization is at least equally plausible.

## Introduction

In psychiatric genetics, the volume of studies examining gene-environment interactions (G x E) is increasing at an accelerating pace [1]. Nonetheless, a growing proportion of these studies appear non-replicable, with clear explanations yet to be found [2–5]. In 2005, Ioannidis responded to surging suspicions of systematic distortion by comparing the ratio of true to non-true simulated relationships, and was able to show that many findings are more likely to be false than true. Moreover, in speculating on “[w]hy most published research findings are false” [6], inconsistencies were coined to reflect bias, partly due to selective publication of the findings [6,7]. Inspired by Ioannidis and colleagues, we aimed to explore multiple testing within individual studies as a plausible explanation for heterogeneity of findings in G x E research on psychiatric disorders.

Ideally, a hypothesis reflects an a priori expectation and the variables involved are operationalized in a single, best possible, way. Subsequently, the test result indicates whether or not the null hypothesis should be rejected. In practice, however, this process usually takes a different form [6,8,9]. In the current era of big data, G x E researchers can often operationalize their genetic, environmental and outcome factors in multiple ways, at least some of which will yield insignificant results. One or more significant G x E findings are to be expected as well: the risk of a false positive (i.e. incorrectly rejected null hypothesis) rises exponentially when testing multiple models with the commonly used hypothesis testing approach. That is, the risk can capitalize from the commonly accepted error chance of 5% in a single test, to over 50% when exploring fifteen or more models (family-wise error: 1-0.95^15^ ≈ 0.54; see, for example: [10]).

Consistent lines of evidence are essential for understanding G x E pathways to psychiatric disorders and advancing the field, but career benefits of reporting significant findings make it conceivable that published associations reflect a non-representative selection of all tests conducted [6,9]. Importantly, this downside of the availability of multiple operationalizations of single factors could be turned into a major advantage if researchers reported all options tried, rather than a biased selection. We postulate that a more transparent overview of all test results could not only reduce publication bias, but also help to move beyond the statement that “most research findings are false” [6] to an approach that allows fellow researchers in the field to consider alternative explanations for ambivalent findings.

This more transparent approach will be illustrated using the interaction between the serotonin-transporter-linked promotor region (*5-HTTLPR*) and childhood adversities with regard to depression. After Caspi and colleagues [11] reported that childhood adversities had the most detrimental effects on depression among those who carried the *5-HTTLPR* short allele, studies exploring this interplay rapidly accumulated. Similar to other genotypes in psychiatric genetics research, however, the original findings of Caspi and colleagues [11] often appeared non-replicable, and even meta-analytical efforts have failed to detect converging lines of evidence [4,12–14].

The exceptional amount of effort devoted to this interaction provides an excellent opportunity to consider possible causes of research equivocality. These can be explored by comparing multiple tests involving varying operationalizations of *5-HTTLPR*, childhood adversities and depression available within a single study. Important explanations for the inconsistent findings are reviewed below, followed by a description of how these were incorporated in the present study.

### Proposed explanations for inconsistent G x E findings

In this section, we describe three commonly acknowledged explanations for inconsistent G x E findings, followed by two explanations that are largely disregarded. First, diverging gene-environment interaction effects are often assumed to arise from differences in the quality of the assessment. More specifically, due to recall bias and interpretation problems of self-report questionnaires, data collected using structured face-to-face interviews conducted by trained interviewers are generally considered more reliable [15]. The lower reliability of self-reports may reduce the power and validity of the interaction tests, possibly explaining why G x E studies using more objective measures have generally yielded more consistent G x E evidence [12,16].

Second, as carriers of genetic risk factors may respond differently to mild and severe childhood adversities than non-carriers, inconsistencies in gene-environment interaction findings may also arise from differences in severity of the childhood adversities examined [17]. With respect to *5-HTTLPR*, the original Caspi et al. [11] study showed a significant moderation by genotype of both the link between childhood maltreatment and depression and the link between milder stressful life events and depression. In contrast, replication studies focusing on severe childhood adversities reported more often significant gene-environment interaction effects than studies involving milder events [12,18], indicating that the probability of significance may co-vary with the severity of the childhood adversity under study.

Third, it may be that individuals are at higher risk of developing psychopathology if they were exposed to adversities in the early stages of their lives [19,20]. That is, effects of environmental adversity on later outcomes may depend on the timing of the adversity [21]. Temporal differences in programming effects of stress on the brain have been proposed because brains are more receptive to stress before the age of five [22]. Programming effects in turn, would render individuals who were exposed to adversities within the first five years of life more prone to depression later on [23], especially when they carry risk alleles [24]. The divergence in G x E findings may thus be due to the difference in the time frame of the adversity measure.

While several studies have addressed these issues, only few researchers have suggested that inconsistencies in G x E research may arise from differences in sex distribution of the sample [25]. There are good reasons to believe that effects of *5-HTTLPR* vary as a function of sex [26], because male and females differ in crucial aspects of the serotonergic neurotransmission encoded by allelic variation in the *5-HTTLPR* gene [27], and the effects of serotonin are increased by the presence of female hormones (i.e. estrogens such as estradiol) [26]. Despite the existence of sex-specific mechanisms, most G x E studies involving *5-HTTLPR* have used mixed samples—implicitly assuming no sex-specific mechanisms—and sex-related heterogeneity in findings has commonly been disregarded (but see [16,28]).

Lastly, inconsistencies in G x E research may also be explained by diverging ways of genotype-coding. The *5-HTTLPR* genotype encompasses a short (S) allele and a long (L) allele variant, which can be modeled in various ways, each based on a different assumption on the relative risk associated with the allelic combinations. Whereas the additive genetic model assumes that the risk increases linearly with the number of S alleles, the dominant model assumes that carriers of the S-alleles have equal risks, regardless of whether their genotype is hetero- or homozygous, and the co-dominant model allows any association pattern without a priori assumptions. Furthermore, in addition to the traditional, so-called biallelic approach of modeling, a triallelic approach has become increasingly popular. The triallelic approach is based on the notion that the L allele is functionally equivalent to the S allele in the presence of the *g* allele variant of the Single Nucleotide Polymorphisms (SNP) rs25531 (*L*_g_ = *S*; see for more details: [29]). Whereas researchers seem to agree upon using the triallelic approach when possible, so far, justification of which genetic model is to be preferred over the others is inconclusive [16,28,30,31]. Although the choice of genetic model may affect association patterns [25,28], several studies found similar effects for different *5-HTTLPR* classifications [32–34].

### This study

In response to suspicions of publication bias among many, and inspiring findings by a few (e.g. [6,35–38]), we examine chance capitalization as a possible explanation for heterogeneity in G x E research. In the remainder of this paper we evaluate the explanations for equivocal G x E findings proposed so far, including chance capitalization, within a single study. Under the assumption that the aforementioned methodological explanations for heterogeneity in G x E research are correct, subsets of regression models containing different measures of childhood adversities and depressive symptoms should yield varying percentages of significant effects.

Using chance capitalization as the competing hypothesis, we expected a significant interaction between *5-HTTLPR* and childhood adversities in more than 5% of the analyses, and especially in the subsets involving: 1) measures based on structured interviews instead of self-report questionnaires; 2) measures of abuse instead of milder childhood adversities; and 3) measures of early adversities instead of those experienced at a later age. Furthermore, we expected the percentages of significant effects to be equal across 4) sex and 5) varying genetic models of *5-HTTLPR*. Absence of meaningful association patterns, with randomly distributed significant effects in, on average, approximately 5% of the analyses, were considered support for publication bias in favor of false positive findings as an explanation for the inconsistent findings reported in the literature [6].

## Method

### Sample

The data were collected as part of the TRacking Adolescents’ Individual Lives Survey (TRAILS), a prospective cohort study of Dutch adolescents [39,40]. Five assessment waves have been completed to date, of which the present study uses data of the first four waves that ran from March 2001 to July 2002 (T1), September 2003 to December 2004 (T2), September 2005 to August 2007 (T3), October 2008 to September 2010 (T4). The study was approved by the Dutch Central Committee on Research Involving Human Subjects (CCMO). Participants were treated in compliance with APA ethical standards, and all measurements were carried out with their adequate understanding and written consent.

At T1, 2230 (pre-)adolescents were enrolled in the study (response rate 76%, mean age 11.1, *SD* = 0.6, 51% girls) [41], of whom 96% (*N *= 2149, mean age 13.6, *SD* = 0.5, 51% girls) participated at T2. The response rates at T3 and T4 were, respectively, 81% (*N *= 1816, mean age 16.3, *SD* = 0.7, 52% girls) and 83% (*N *= 1881, mean age 19.1, *SD* = 0.6, 52% girls). The data collection included, among many other things, self-report and parent-report questionnaires, a psychiatric diagnostic interview, and a life stress interview.

### Measures

#### Depressive symptoms

Depressive symptoms were assessed using three different instruments, which yielded nine measures in total (for an overview, see Table 1). Participants’ parents reported on depressive symptoms by means of the 13-item DSM-oriented Affective Problem Scale [42] of the Child Behavior Checklist (CBCL; [43]), the participants themselves reported on depressive symptoms by its self-report variant, the 13-item Affective Problem Scale of the Youth Self-Report (YSR; [39]). Both instruments assessed depressive symptoms over the last six months, with possible answers 0 = not true, 1 = somewhat true or sometimes true, and 2 = very true or often true. The CBCL and YSR were assessed at T1 (CBCL_1_, α = .68; YSR_1_ α = .77), T2 (CBCL_2_, α = .73; YSR_2_ α = .72) and T3 (CBCL_3_, α = .76; YSR_3_, α = .78). In addition to the scores for each assessment wave separately, we calculated the mean over these three time points, labeled as CBCL_mean_ and YSR_mean_, respectively.

**Table 1:** Descriptive statistics of the variables used in the study.

					*Males*		*Females*		*Total*	
	Resp.	Instrument used / genetic approach	Age	label	Mean	SD	Mean	SD	Mean	SD
**Depressive symptoms**	Parent	Child Behavior Checklist (CBCL)	11	CBCL_1_	0.20	0.20	0.18	0.19	0.19	0.20
	Parent	Child Behavior Checklist (CBCL)	13	CBCL_2_	0.15	0.19	0.15	0.19	0.15	0.19
	Parent	Child Behavior Checklist (CBCL)	16	CBCL_3_	0.14	0.18	0.17	0.22	0.16	0.21
	Parent	Child Behavior Checklist (CBCL)		CBCL_mean_	0.16	0.16	0.16	0.16	0.16	0.16
	Self	Youth Self Report (YSR)	11	YSR_1_	0.29	0.25	0.30	0.25	0.29	0.25
	Self	Youth Self Report (YSR)	13	YSR_2_	0.22	0.22	0.32	0.29	0.27	0.26
	Self	Youth Self Report (YSR)	16	YSR_3_	0.22	0.22	0.36	0.30	0.30	0.27
	Self	Youth Self Report (YSR)		YSR_mean_	0.25	0.19	0.32	0.23	0.28	0.21
	Self	CIDI, lifetime prevalence Major Depressive Episode		CIDI	6.9	(*n* = 76)	16.8	(*n* = 190)	11.9	(*n* = 266)
**Childhood adversities**	Parent	Questions on pre- & perinatal risks	>1	_PP_RISKS	1.45	1.03	1.41	1.08	1.43	1.05
	Parent	TRAILS Family History Interview on Childhood Events	>11	CE	0.70	0.83	0.73	0.86	0.71	0.84
	Parent	Questions on Long Term Difficulties	>11	LTD	0.45	0.72	0.36	0.65	0.40	0.69
	Parent	Subjective ratings of the stressfulness of the participant’s life during childhood	>5	_parent0-5_STRESS	1.62	2.03	1.54	2.07	1.58	2.05
	Parent	Subjective ratings of the stressfulness of the participant’s life during childhood	6–11	_parent6-11_STRESS	2.39	2.35	2.48	2.48	2.43	2.42
	Self	Subjective ratings of the stressfulness of the participant’s life during childhood	>5	_self0-5_STRESS	2.52	2.06	2.32	1.86	2.42	1.96
	Self	Subjective ratings of the stressfulness of the participant’s life during childhood	6–11	_self6-11_STRESS	3.48	2.29	3.28	2.26	3.37	2.28
	Self	Childhood Trauma Questionnaire (CTQ)	>16	_verbal_ABUSE	1.69	0.69	1.79	0.80	1.74	0.76
	Self	Childhood Trauma Questionnaire (CTQ)	>16	_phys_ABUSE	1.18	0.32	1.19	0.36	1.18	0.34
	Self	Childhood Trauma Questionnaire (CTQ)	>16	_sex_ABUSE	1.03	0.17	1.09	0.33	1.06	0.27
**Genotype**	SS/ S'S'				17.9	25.2	16.5	23.6	17.2	24.4
	LS/ L'S'				45.2	48.1	48.3	51.7	46.8	50.1
	LL/ L'L'				36.9	26.7	35.2	24.7	36.0	25.6

Note: Resp. (column 2) refers to the type of informant or, for the *5-HTTLPR* genotype, to the allele; Age (column 3) reflects the approximate age at measurement if relevant; with regard to the CIDI and *5-HTTLPR*, instead of means and SD, the descriptive statistics denote prevalence percent with frequency in brackets. With regard to genotypes, instead of means and SD, the descriptive statistics denote prevalence percent for the biallelic (i.e. excluding SNP rs25531) and triallelic (i.e. including SNP rs25531) approach respectively. For exact computation of genetic models, see supplementary material.

The presence of a lifetime Major Depressive Episode according to the Diagnostic and Statistical Manual of Mental Disorders (DSM-IV; [44]) was assessed at T4, using the World Health Organization Composite International Diagnostic Interview (WHO CIDI; version 3.0). The CIDI has been used in a large number of surveys worldwide [45], and shown to have good concordance with clinical diagnoses [46,47].

#### Childhood adversities

Exposure to childhood adversities was operationalized in ten different ways (for an overview, see Table 1). ‘Pre- and perinatal risks’ (_PP_RISKS) were assessed at T1 in an interview with one of the parents, usually the mother, and included questions about maternal prenatal smoking, maternal prenatal alcohol use, birth weight, gestational age, and pregnancy and delivery complications. Maternal prenatal smoking was coded as follows: 0 = no smoking; 1 = 10 cigarettes a day or less; 2 = more than 10 cigarettes a day. Maternal prenatal alcohol use was coded as follows: 0 = no alcohol use, 1 = up to three glasses per week, 2 = four glasses per week or more. Birth weight was coded as 0 = normal birth weight and 1 = either low (< 2,500 g) or high (> 4,500 g) birth weight. Gestational age was recoded into two groups: 0 = normal (between 34 and 42 weeks) and 1 = abnormal (33 weeks or less, or more than 42 weeks). Pregnancy and delivery complications were coded as 0 = no complications; 1 = between one and four complications; 2 = five or more complications. The index of pre- and perinatal risks was composed by adding the scores of these variables.

The measure‘Childhood events’ (CE) reflects stressful events that occurred before T1, and was also assessed at the T1 parent interview. The variable was created by summing the following events: severe (physical or mental) disease of father or mother; severe illness (life-threatening or threat of serious permanent effects) of a sibling; death of a household member; parental divorce; and absence from home for three months or longer.

‘Long term difficulties’ (LTD) were assessed with a questionnaire completed by parents at T2, and reflects the sum score of the following difficulties: chronic disease or handicap of the participant, chronic disease or handicap of a household member, being bullied, long-lasting conflicts with a household member, and long-lasting conflicts with someone else, all experienced before T1.

Four measures involved subjective ratings of the stressfulness of the participants’ lives during early and later childhood. These were assessed at T2 using parent- and self-reported ratings of the overall stressfulness of the child’s life before the age of five (_parent0-5_STRESS and _self0-5_STRESS) and from age six to age eleven (_parent6-11_STRESS and _self6-11_STRESS). Parents were asked ‘How stressful was your child’s life in this life phase?’, and the participants ‘How many stressful events did you experience in this period?’ The stressfulness was rated on an 11-point scale, ranging from 0 = not at all to 10 = very much.

Three measures represented traumatic youth experiences before the age of sixteen years, as reported retrospectively by the adolescents at T4, with questions based on the Childhood Trauma Questionnaire (CTQ; [48]). Verbal abuse (_verbal_ABUSE, α = .84) was measured by five questions ranging from screaming to threatening and physical abuse (_phys_ABUSE, α = .73) by six questions ranging from being hit to being beaten up; the occurrence of which could be rated as 1 = no, never; 2 = yes, one or two times; 3 = sometimes; 4 = often; 5 = very often. Sexual abuse (_sex_ABUSE) was based on a list of five unwanted sexual acts by an adult family member, friend of the family or stranger, ranging from touching to sexual intercourse, the occurrence of which could be rated as 1 = never happened to me, 2 = happened once, or 3 = happened several times. The abuse measures reflect the mean of the ratings.

#### 5-HTTLPR

DNA was obtained at T3 through a manual salting out procedure using either blood samples or buccal swabs, as delineated in detail by Miller, Dykes, and Polesky [49]. The length polymorphisms of the serotonin transporter-linked polymorphic region (*5-HTTLPR*) were genotyped as described by Nederhof and colleagues [50].

The *5-HTTLPR* polymorphism was modeled in an additive, dominant and co-dominant way, which, combined with the biallelic and triallelic approach, yielded six different ways to operationalize genetic risk. In the additive genetic model, the risk associated with the LS genotype was coded as being half the size as the risk associated with the SS genotype, as compared to the LL genotype (LL = 0, LS = 1, SS = 2). In the S dominant model, the risk associated with the of SL and SS genotype was modeled as being equal to each other (LL = 0, LS = 1, SS = 1). In the co-dominant model, the risk associated with SS and LS was estimated using separate dummies, with LL serving as the reference group for both (dummy 1: LL = 0, LS = 0, SS = 1; dummy 2: LL = 0, LS = 1, SS = 0). These three genetic models were constructed using both the biallelic approach and the triallelic approach. In the latter case, the L allele was recoded into an S allele in the presence of ag allele of SNP rs25531 (Lg = S). For an overview of *5-HTTLPR* coding in regression models per genetic model, see (S1 Table). Please bear in mind that, due to the dummy coding as dictated by the co-dominant model, the six genetic models were in the statistical analyses tested by a total of eight interaction terms.

### Analytical strategy

We tested a total of 2160 interaction effects between *5-HTTLPR* and childhood adversities on depressive symptoms, using ordinary least squares (for continuous outcomes) and logistic regression (for binary outcomes) in SPSS (version 21; [51]). The tests were conducted in a sample comprised of both males and females (See Table 2 for an overview of all analyses; 720 regression models, sample sizes ranging from 779 to 1050), a male sample (720 regression models; sample sizes 341 to 488), and a female sample (720 regression models; sample sizes ranging from 438 up to 562). Each analytic model included one of the childhood adversity measures, one of the operationalization’s of *5-HTTLPR* (in case of co-dominant genetic models: two dummy variables), and one of the depression measures as outcome variable. We were primarily interested in the interaction between the childhood adversity and the *5-HTTLPR* measure.

**Table 2:** Percentage of significant (*p* < .05) G x E interaction effects per subset of analyses.

Relevant subsets	Subset 1	Total tests		Nr Sign		Subset 2	Total tests		Nr Sign		Subset 3	Total tests		Nr Sign	
Interview (subset 1) versus questionnaires (subset 2)	_PP_RISKS; CE; CIDI	224	(842–1050)	1	**(0.4%)**	LTD; _self0-5_STRESS;_self6-11_STRESS; _parent6-11_STRESS; _self6-11_STRESS; _verbal_ABUSE; _phys_ABUSE; _sex_ABUSE; CBCL_1; 2; 3_; CBCL_mean_; YSR_1; 2; 3_; YSR_mean_	1216	(779–1050)	77	**(6.3%)**					
Severe adversity (subset 1) versus mild adversity (subset 2)	_verbal_ABUSE; _phys_ABUSE; _sex_ABUSE	216	(779–939)	4	**(1.9%)**	_parent0-5_STRESS; _self0-5_STRESS; _parent6-11_STRESS; _self6-11_STRESS	288	(828–1034)	27	**(9.4%)**					
Early adversity (subset 1) versus late adversity (subset 2)	_parent0-5_STRESS; _self0-5_STRESS	144	(828–1031)	13	**(9.0%)**	_self6-11_STRESS; _parent6-11_STRESS	144	(829–1034)	14	**(9.7%)**					
Males (subset 1) versus female (subset 2)	Males	720	(341–488)	264	**(12.2%)**	Females	720	(438–562)	132	**(6.1%)**					
*5-HTTLPR*, biallelic (subset 1) versus triallelic (subset 2)	Biallelic	360	(779–1050)	26	**(7.2%)**	Triallelic	360	(779–1050)	13	**(3.6%)**					
*5-HTTLPR*, dominant (subset 1) versus co-dominant (subset 2) versus additive (subset 3)	Dominant	180	(779–1050)	7	**(3.9%)**	Co-dominant	360	(779–1050)	18	**(5.0%)**	Additive	180	(779–1050)	14	**(7.8%)**

Note: ‘Total Tests’ reflects number of total tests run, with in brackets the minimum and maximum sample size over all single tests conducted; ‘Nr Sign’ refers to number of significant findings by single tests (*p* < .05), with significance rate over the relevant measures in brackets (i.e. number of significant findings relative to the total number of tests run). For meaning of abbreviations within each subset of measures, see Table 1.

In order to explore our hypotheses, we calculated the percentage of significant findings for subsets of regression analyses, by dividing the number of statistically significant (*p* < .05) interaction effects by the total number of interaction effects tested, and multiplying this proportion by 100. Because the tests could be considered neither independent observations nor repeated observations, formal testing of the hypothesis that the percentage of significant interactions (in a given subset) exceeded chance level (i.e., 5%) was not possible. Nevertheless, to provide a guideline to determine which percentage can be considered indicative of a real interaction effect we calculated a confidence interval around 5%.

The number of tests in this study ranged between 144 and 1216 with an average of almost 400 (see Table 2). The tests conducted were clearly dependent, and were therefore considered not to reflect 400 independent repetitions, but considerably fewer. Given the substantial dependence among the tests (same sample, and partly same measures) and the fact that the number of tests performed varied widely around an average of 400, we chose a conservative approach and based the calculation on 200 independent tests. In this hypothetical situation, the upper bound of the confidence interval would be 8% (5% ± 1.96 * √ (5 * 95) / 200). In other words, we considered more than 8% significant interactions a fair indication that the percentage exceeded the Type 1 error rate dictated by the .05 alpha, and that the interaction as operationalized in the particular subset may actually exist in the population. In addition to evaluating the percentage of significant interactions, we visually inspected the distribution of significant findings by means of a plot that depicted the number of significant finding for each of the possible interaction pathways. The absence of particular (non-random) patterns was regarded as random distribution of findings, and indicative of a significant presence of chance findings.

## Results

### Interview versus questionnaire

When comparing subsets of regression models that contained either interview-based or questionnaire-based measures, we found more significant interaction effects when using questionnaire-based measures (see Table 2). Whereas G x E interaction tests involving interview-based measures yielded 0.4% significant interaction effects, tests based on questionnaires generated 6.3% significant interactions. None of these percentages exceeded the preset criterion of at least 8% significant effects; the number of interview-based significant interactions was even below what might be expected on the basis of chance.

### Severe versus mild adversity

Of all interaction effects containing severe childhood adversities 1.9% reached statistical significance. In contrast, inclusion of mild childhood adversities yielded 9.4% significant interactions (see Table 2), and therewith exceeded the preset 8%-criterion.

### Early versus late adversity

Table 2 further shows that 9.0% of the interaction effects involving early childhood adversities, and 9.7% of those involving adversities in later childhood reached statistical significance, both of which were higher than what might be expected based on chance. Note that these two subsets both related to mild adversities, which had an overall significance prevalence of 9.4%.

### Males versus females

While the 720 analyses run in only female participants yielded 6.1% significant interactions, the same analyses run in male participants yielded 12.2% (see Table 2). Only the percentage in males exceeded the preset criterion of 8%.

### Genetic models

As depicted in Table 2, regression models with *5-HTTLPR* coded either in accordance with the biallelic or triallelic statistical approach, yielded 7.2% and 3.6% significant interactions, respectively. In addition, we found 3.9% significant interaction effects when testing models containing the dominant genetic model of *5-HTTLPR*, whereas those containing the co-dominant or additive genetic models yielded respectively 5.0% and 7.8% significance. None of these percentages were higher than the preset criterion of 8%.

### Chance capitalization

**Fig 1:**
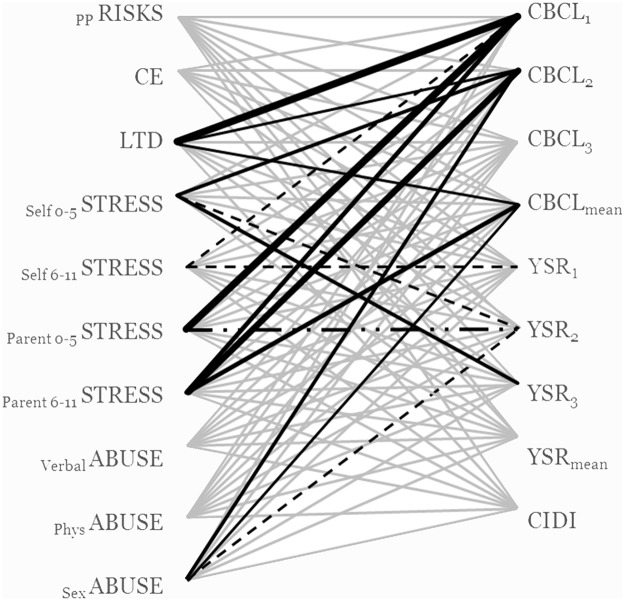
Number of significant interaction effects per combination of measures. Each line represents a possible interaction pathway between the ten childhood adversities (labels depicted on the left) and *5-HTTLPR* genetic risk (i.e. additive, dominant and co-dominant way, which, combined with the biallelic and triallelic approach, yield six different ways to operationalize genetic risk) on depressive symptoms measured by the nine different instruments (labels depicted on the right side of the figure). Grey lines represent insignificance Black lines represent the number of significant interaction effects (*p* < .05) over six different ways to operationalize genetic risk: the bolder the black line, the higher the number of significant effects for that specific combination of measures. Solid black lines represent significant interactions by the SS genotype; dotted black lines represent significant interactions by LS genotype. Partially dotted lines represent significant interaction(s) of S-carriers. *N *= 779–1050, dependent on the combination of measures (see Table 2).

Overall, from the 2160 number of interaction effects tested, 171 were significant (*p* < .05), corresponding with a 7.9% overall significance rate. When ignoring the analyses regarding early versus late adversities (because these were nested within the subset of mild stressors), two subsets of analyses generated a percentage of significant interaction that exceeded the preset criterion for substantial elevation above chance level (8%), whereas two others yielded a percentage of significant interactions that was substantially below what might be expected on chance (< 2%). Fig 1 shows that predominantly parent-reported long-term difficulties (LTD), parent-reported overall stressfulness of the child’s life (_parent0-5_STRESS; _parent6-11_STRESS), and parent-reported depressive symptoms (CBCL), seem to yield the highest number of significant interaction effects with *5-HTTLPR*.

## Discussion

Unraveling the genetic basis of psychiatric disorders appears to be challenging, and non-replications have become increasingly common in this area of research, particularly with regard to gene-environment interactions (G x E). Ioannidis [6] postulated chance capitalization as the main contributor to inconsistencies in G x E research. Starting from this premise, we evaluated the plausibility of alternative explanations, by examining patterns of significance related to specific methodological choices within a single dataset. In addition to chance capitalization, we explored the option that inconsistencies in G x E findings arise from differences in assessments, severity of the adversity, and timing of the adversity. Heterogeneity due to differences in the sex distribution of the sample or genetic model used was also explored. The database of the Dutch cohort study of adolescents TRAILS contains various different measures of childhood adversities and depressive symptoms in addition to genotypic information, and was hence suitable for exploring the often-described interaction between *5-HTTLPR* and childhood adversities in predicting depressive symptoms.

With regard to the quality of assessments, we expected more significant interaction effects in analyses involving interview-based than in analyses involving questionnaire-based measures, but found the opposite pattern. Questionnaire-based measures, although assumed to be less reliable than structured face-to-face interviews assessed by trained interviewers [15], might thus be of less importance in explaining G x E heterogeneity than commonly assumed. Although, in our sample, questionnaire-based measures performed better than interview-based measures in terms of percentage of significant interactions, the number of significant interactions exceeded our preset 8%-criterion of what might be expected based on merely chance by 1.3%. This seemingly contradictory result, however, could also be explained by measurement properties and its associated power. That is, the high percentage of significance found for questionnaires was predominantly determined by significant findings in models based on scales (i.e. CBCL; 29 of the total 77), but also models based on single-item questionnaire variables (i.e. parent- and self-reported ratings of the overall stressfulness; 27 of the total 77). The higher number of significant findings for questionnaires may thus be partly explained by the lack of power of the dichotomous variable.

The significance rates in analyses that differed with respect to the severity of the childhood adversities were also contrary to what we expected based on prior findings, which suggest a higher probability of significance in case of severe adversities such as abuse [12,18]. Our analyses indicated the opposite: more significant interactions in models containing measures of mild childhood adversity than in those containing measures of abuse. Importantly, mild adversities were also far more common in TRAILS than more severe adversities, and the larger percentage of significant results may reflect the actual distribution of scores.

While it has been proposed that adversities experienced at an early age have more developmental impact than those experienced later in childhood [19,20,24], the timing of the adversities did not affect the number of significant G x E interactions in our sample. This suggests that the assumed timing effects cannot explain differences among studies with regard to their interaction with *5-HTTLPR*.

We found differences in significance rates as a function of sex. Although effects of *5-HTTLPR* on serotonergic neurotransmission possibly explain differences between males and females [26], only few authors have pointed out sex-specific mechanisms as possible reasons for heterogeneity [16,25,28]. Given that significant effects were found for males in particular, the difference may be due to hormone-driven sex differences in brain and behavior. That is, although sex differences are often disregarded, in females, serotonin may be conditional on the presence of female estrogens such as estradiol to exert its full effect [26]. The absence of female hormones before puberty could explain why significance rates were twice as high in males as in females, as TRAILS participants were approximately 11 to 19 years old. Across G x E studies, findings may thus co-vary by age differences across samples, at least for *5-HTTLPR*, childhood adversity and (pre)adolescent depression.

Finally, and again contrary to what we expected a priori, different genetic models yielded varying percentages of significance, and the biallelic approach seemed to yield more significant interactions than the triallelic approach. However, none of the genetic models yielded significance rates that substantially exceeded the a priori set criterion of 8%, so our study does not provide enough evidence to abolish the notion that the choice of genetic model does not influence the probability of significance [16,28,31].

### Methodological differences or chance capitalization?

As described above, we explored the consequences of a number of methodological differences among studies that have been proposed to explain inconsistencies in the field of G x E research, in this case the interaction of childhood adversities and *5-HTTLPR* with regard to depressive symptoms. The alternative hypothesis postulated chance capitalization to explain heterogeneity in findings [6]. In order to abandon the idea of chance capitalization as important contributor to the lack of consistent findings, the percentage of significance findings should be clearly higher than what would be expected on the basis of chance (about 8% in the present study) in at least some analyses. Interestingly, the overall percentage of significant interactions of 7.9% came close to the predefined threshold for an elevated significance rate of 8%. Still, it did not exceed this threshold, which makes it plausible that at least part of the heterogeneity in gene-environment interactions results from overpublication of false positive findings. The 8% criterion used is somewhat arbitrary but comparable to commonly used criteria to denote, for example, a ‘large’ or ‘clinically relevant’ effect. If we had based the calculations on 400 tests instead of 200, the criterion for considering a percentage clearly exceeding 5% would have been 7.1% and the conclusion on the likely presence of a gene by environment interaction would have been affirmative. The findings should therefore be interpreted with caution.

In some analyses, the percentage of significant interactions exceeded the threshold of 8%, but in others the significance rate found was lower than 5%. In other words, the percentages were somewhat randomly distributed around chance level. Moreover, the characteristics of the analyses that yielded relatively high significance rates did not correspond to what was expected in advance, which further support the idea of random fluctuations.

In line with earlier findings [38], our results suggest that at least part of the heterogeneity in gene-environment interactions reported is due to overpublication of false positive findings. Nonetheless, differences in sampling and measurement error may represent alternative explanations for the observed variability of results in the G x E literature, and our observation is too tentative to determine whether inconsistencies in the field of G x E research are explained by methodological differences or chance capitalization.

### Strengths and limitations

Our study has various strengths. First and foremost, we are among the first to provide a transparent overview of the many possible ways to operationalize a specific gene-environment interaction within a single cohort study and to actually test all these interactions in order to explore ways to deal with scientific threats like multiple testing and selective reporting. Beyond qualifying multiple testing as a mere methodological issue that can lead to chance capitalization, we illustrate the potential benefits of multiple tests in understanding ambivalence in the psychiatric genetics literature. A second strength is that our interaction tests were each based on a relatively large number of individuals, offering reasonable power and accuracy—at least for the total sample. That is, while part of the gender-specific regression analyses may have been underpowered because some outcome variables were dichotomous and main effects sizes for G and E depend on the variables used, the analyses using the total sample were sufficiently powered.

The study is also limited in a number of respects. First, we illustrated the possible role of chance capitalization by using *5-HTTLPR*. Although threats like multiple testing and selective reporting are likely equal within G x E research, the extent to which chance capitalization contributes to equivocal evidence may differ across genotypes. Second, we relied on a fairly large number of questionnaire-based measures and only few interview-based measures. Moreover, of the three interview-based measures, CIDI and CE may be less powered than continuous questionnaire-based measures. Therefore, replication of these findings in studies with other and preferably more (continuous) interview-based measures is needed.

## Conclusion and Future Prospects

Taken together, our results demonstrate the need for caution in interpreting findings from datasets with comparable measures of the same construct. Obvious career benefits of reporting significant findings over null findings and easy access to comparable measures within one dataset may lead to overpublication of false positive findings. Hence, it is likely that chance capitalization explains at least partly the large number of inconsistencies in G x E research.

Large cohort studies could play a major role in paving ways towards more consistent G x E findings in future research. The availability of multiple operationalizations of single factors, now often disregarded or even suspected as source of selective reporting, could be turned into a major advantage if researchers reported all options tried, rather than a biased selection. This way, we can move beyond the statement that “most research findings are false”, towards a novel approach: critically discussing the range of available measures within each study to evaluate the plausibility of findings, given the likelihood of chance capitalization.

## Supporting Information

S1 Table Overview of *5-HTTLPR* coding in regression models, per genetic model. (DOCX)
