# Correction: I Just Ran a Thousand Analyses: Benefits of Multiple Testing in Understanding Equivocal Evidence on Gene-Environment Interactions

**DOI:** 10.1371/journal.pone.0213750

**Published:** 2019-03-07

**Authors:** Vera E. Heininga, Albertine J. Oldehinkel, René Veenstra, Esther Nederhof

## Notice of republication

This article was republished on February 27, 2019 to remove a Supporting Information file that was incorrectly included in the originally published article. Please download this article again to view the correct version.
