# Supplementary material for: I Just Ran a Thousand Analyses: Benefits of Multiple Testing in Understanding Equivocal Evidence on Gene-Environment Interactions
Source: PLoS One. 2015 May 27;10(5):e0125383. doi: 10.1371/journal.pone.0125383 (PMC4446037; doi:10.1371/journal.pone.0125383)
Supplement: S1 Table — (DOCX) [file pone.0125383.s001.docx]

**S1 Table. Overview of *5-HTTLPR* coding in regression models, per genetic model**

| **Genotype:** |  | **LaLa** | **LaLg** | **LgLg** | **LaS** | **LgS** | **SS** |
| --- | --- | --- | --- | --- | --- | --- | --- |
| *Biallelic coding:* |  | *LL* | *LL* | *LL* | *LS* | *LS* | *SS* |
|  | Additive model: | 0 | 0 | 0 | 1 | 1 | 2 |
|  | Dominant model: | 0 | 0 | 0 | 1 | 1 | 1 |
|  | Co-dominant model: |  |  |  |  |  |  |
|  | - Dummy LS: | 0 | 0 | 0 | 1 | 1 | 0 |
|  | - Dummy SS: | 0 | 0 | 0 | 0 | 0 | 1 |
| *Triallelic coding:* |  | *L’L’* | *L’S’* | *S’S’* | *L’S’* | *S’S’* | *S’S’* |
|  | Additive model: | 0 | 1 | 2 | 1 | 2 | 2 |
|  | Dominant model: | 0 | 1 | 1 | 1 | 1 | 1 |
|  | Co-dominant model: |  |  |  |  |  |  |
|  | - Dummy L’S’: | 0 | 1 | 0 | 1 | 0 | 0 |
|  | - Dummy S’S’: | 0 | 0 | 1 | 0 | 1 | 1 |
